# Comparison of sealer penetration of sonic activation versus conventional needle irrigation: a systematic review and meta-analysis of randomized controlled trials

**DOI:** 10.1186/s12903-022-02608-1

**Published:** 2022-12-03

**Authors:** Li Tan, Qiong Liu, Yun Chen, Ya-Qiong Zhao, Jie Zhao, Marie Aimee Dusenge, Yao Feng, Qin Ye, Jing Hu, Ze-Yue Ou-Yang, Ying-Hui Zhou, Yue Guo, Yun-Zhi Feng

**Affiliations:** 1 Department of Stomatology, The Second Xiangya Hospital, Central South University, Changsha, 410011 Hunan China; 2 National Clinical Research Center for Metabolic Diseases, Hunan Provincial Key Laboratory of Metabolic Bone Diseases, and Department of Metabolism and Endocrinology, The Second Xiangya Hospital of Central South University, Changsha, 410011 Hunan China

**Keywords:** Sonic activation techniques, Root canal therapy, Irrigation, Sealer penetration, Systematic review

## Abstract

**Background:**

Most existing studies comparing the efficiency of sonic irrigation (SI) and conventional needle irrigation (CNI) in increasing the penetration of sealers into dentine tubules are controversial; and this study aimed to determine whether the use of SI can lead to greater sealing ability than CNI, during the root canal treatment.

**Methods:**

The EMBASE, PubMed, and Cochrane Library databases were used to find confocal laser scanning microscopy studies evaluating percentage and maximum depth of sealer penetration following the use of SI or CNI in mature permanent teeth until October 2022. The critical estimative checklist of randomized controlled trials of the standardized Joanna Briggs Institute was adopted to independently score the quality of each study. The random-effect model for meta-analysis was used to analyse for each canal segment (apical, middle, coronal). The results are shown in the forest plots as weighted mean differences (WMDs) with 95% confidence intervals (95% CIs).

**Results:**

Ninety-seven articles were included in the preliminary screening, and nine of them were included in this study. Eight studies were included in the meta-analysis.The meta-analysis exhibited great increases in the coronal (WMD: 8.09, 95% CI 2.78–13.40/WMD: 165.32, 95% CI 128.85–201.80), and middle segments (WMD: 8.81, 95% CI 5.76–11.87/WMD: 132.98, 95% CI 68.71–197.25) for the percentage and maximum depth of sealer penetration, respectively. The percentage of sealer penetration in the apical thirds region was nonsignificant (WMD: 4.73, 95% CI − 2.34–11.80). However, the maximum depth of sealer penetration in the apical thirds region was significant (WMD: 121.46, 95% CI 86.55–156.38). Chi-squared analysis revealed heterogeneity scores of 0.0–70.0% and 44.0–90.0% for the percentage and maximum depth of sealer penetration, respectively.

**Discussion:**

This review verified that SI significantly improves tubular dentin sealer penetration in most areas of the root canal; thus, SI may lead to better filling efficiency and anti-reinfection effects than CNI during and after the root canal therapy. Nevertheless, a large heterogeneity in the current data comparing the irrigation efficiency of SI versus CNI in the apical third of the root canal was found, implying the necessity to standardize root canal irrigation procedures and obtain more accurate results in this area.

*Trial Registration*: INPLASY database (INPLASY202270116).

**Supplementary Information:**

The online version contains supplementary material available at 10.1186/s12903-022-02608-1.

## Background

The three-dimensional filling of root canals during the whole process of root canal therapy is very important and can significantly increase the success rate of this treatment [1, 2]. Sealing dentinal tubules with sealers can not only improve the filling efficiency of the root canal by providing better adhesive strength between the filling material and dentin wall due to chemical and micromechanical bonding of sealers and dentinal tubules [3–7], but also reduce the possibility of reinfection after root canal treatment by preventing bacteria from entering dentinal tubules or burying residual microorganisms in the dentinal tubules [8]. However, mechanical instrumentation of root canal therapy can produce a residual smear layer that can adhere to the surface of dentin tubules and prevent sealers from entering the dentin tubules [9]. Therefore, to effectively clean out the smear layer and improve the permeability of sealers to dentinal tubules, many irrigation techniques have been developed in clinical practise [10–12].

Conventional needle irrigation (CNI) was the earliest and is most convenient irrigation strategy applied in the root canal treatment [13]. However, the irrigation efficiency of CNI cannot perfectly meet the clinical demands. Because it is difficult to deliver irrigation solutions into intricate areas of root canals, such as the apical third region with CNI, gas particles can become entrapped to produce a vapor lock effect [14, 15]. Therefore, clinicians invented sonic activation (SI) techniques with the aim of overcoming the shortcomings of CNI [16].

Although there are a large number of reports comparing the efficiency of SI and CNI in sealing dentinal tubules with sealers, outcomes are often conflicting [10, 17, 18]. Until now the problem of whether SI would produce more favourable results in sealer penetration than CNI had not been analysed by a meta-analysis. Therefore, it is meaningful and necessary to conduct a summative and evidence-based review of the current study results in this area.

First, this study aimed to determine whether the use of SI can lead to greater sealing ability than CNI during the root canal treatment. Then, the meta-analysis focused on the sealing ability of SI at different depths of the canal. The tested null hypothesis was that the difference in the sealing ability between CNI and SI is not remarkable.

## Methods

The protocol of this study has been registered in the INPLASY platform (INPLASY202270116), and this article followed the PRISMA 2020 statement (Page et al. 2020). The PRISMA 2020 checklist and PRISMA 2020 abstract checklist were also uploaded as Additional file 3: Table S1 and Additional file 4: Table S2.

### Eligibility criteria

One study showed that the percentage and maximum depth of sealer penetration can perfectly reflect the three-dimensional sealing ability of sealers [19]. Some other studies also shows that the percentage of sealer penetration is a more clinically relevant parameter than other parameters for indication endodontic seal quality [10, 18]. Therefore, we chose these two common indicators to study the sealing ability of sealers. Then, a thorough search was conducted for all previous studies assessing the efficacy of the percentage and maximum depth of sealer penetration, following the use of SI (all sonic systems that meet the inclusion criteria will be included) and standardized irrigants (NaOCl and EDTA). Because tubular dentin sealer penetration can hardly be measured clinically, and confocal laser scanning microscopy (CLSM) studies is widely used for evaluating penetration, only studies using CLSM were chosen for this analysis. Studies using samples that were filled roots or nonhuman teeth, artificial debris, and plastic blocks, and studies measuring the penetration of tubular dentin sealers in the lateral branch of the root canal, isthmus, or artificial grooves were excluded to maintain the standardized sample selection and measurement [20]. The publication dates of the article was limited to between January 2010 and October 2022 to ensure that conclusions were drawn from contemporary data. There were no language restrictions on filtering articles to ensure the integrity of the included data.

### Information sources

In October 2022, three well known databases (PubMed, EMBASE, Cochrane Library) related to previously published studies in endodontia were screened. Furthermore, the references of screening studies and the 2022 edition of the journals related to Endodontics (AEJ, IEJ, JOE) were searched manually.

### Search

The search was based on the following PICO framework:(P) the tooth taken only for inclusion; (I) using SI for root canal irrigation; (C) using CNI for root canal irrigation; and (O) the penetration efficiency of sealers into dentine tubules which was assessed by CLSM.

A focused search strategy was developed with a combination of MeSH (medical subject headings) terms and key terms which was related to the topics of ‘root canal’,‘sonic irrigation’,‘conventional needle irrigation’ and ‘tubular dentin sealer penetration’. After that, the author's knowledge, literature and index database are used to identify and expand upon these headings through synonyms, key words and index words. Finally, a search strategy using Boolean and truncation operators (‘OR’, ‘AND’) was implemented, which gave consideration to sensitivity and specificity, and adjusted for each database, e.g., the Pubmed search strategy is shown in Table 1. (The search strategy used for EMBASE and cochrane was uploaded as Additional file 5: Tables S3 and Additional file 6: Table S4.)

**Table 1:** PubMed search strategy

PubMed	Search Strategy (October, 2022)	Items
#1	"dentinal tubules"[Title/Abstract] OR "root canal"[Title/Abstract] OR "root canals"[Title/Abstract] OR "root dentine"[Title/Abstract] OR "dentinal tubule"[Title/Abstract] OR "premolars"[Title/Abstract] OR "tubules"[Title/Abstract] OR "Dentition"[Title/Abstract] OR "dentition, permanent"[MeSH Terms] OR "incisor"[MeSH Terms] OR "bicuspid"[MeSH Terms] OR "cuspid"[MeSH Terms]	119,521
#2	"sonic irrigation"[Title/Abstract] OR "endoactivator"[Title/Abstract] OR "sonication"[Title/Abstract] OR "EDDY"[Title/Abstract] OR "sonic activation"[Title/Abstract] OR "sonication"[MeSH Terms]	19,798
#3	"sealer penetration"[Title/Abstract] OR "depth of penetration"[Title/Abstract] OR "penetration depth"[Title/Abstract] OR "sealer penetration"[Title/Abstract] OR "Sealing"[Title/Abstract] OR "tubule penetration"[Title/Abstract] OR "dentin permeability"[MeSH Terms]	19,622
#4	#1 AND #2 AND #3	33

### Study selection

Duplicate articles were detected by using EndNote 20 (Clarivate Analytics, Philadelphia, Pennsylvania, USA) software and deleted manually by the author. After removing repetitive articles, two checkers (QL & YC) evaluated the titles/abstracts and full-text independently by using the above criteria to select appropriate studies. In case of any dispute between the two reviewers, the third reviewer (YQ-Z) participated in the discussion and resolved the disagreements. For quality evaluation and evidence synthesis, the data of the selected studies were extracted by the same reviewer using a standardized prepiloted form.

### Items of data

The reviewed and collected data involved items about the group design, tooth types, needle and tip sizes of CNI and SI, materials for closing the canal system, concentration of irrigant, instrumentation system, surgical diameter, type of sealer, CLSM magnification, outcomes for percentage and maximum depth of sealer penetration in the coronal, middle and apical regions of canals. All of the above imformations is summarized in Table 2.

**Table 2:** Methodological characteristics and critical appraisal of all studies included

Studies	Sample	Closed system	Irrigants	CLSM magnification	Groups design(n)	Canal segments	Instrumentation system	Surgical diameter	Type of sealer
Akcay et al. [30]	Mandibular premolars	Wax	5.0% NaOCl & 17% EDTA	× 10	SI(13), CNI(13)	A&M&C	Pro Taper	F4	AH Plus^a^
Ateş et al. [24]	Mandibular incisors	Cyanoacrylate	5.0% NaOCl & 17% EDTA	× 5	SI(16), CNI(16)	A&C	Xpress fles	30/.04	BC Sealer^b^
Bharti et al. [25]	Mandibular premolars	Wax	3.0% NaOCl & 17% EDTA	Unknown	SI(10), CNI(10)	A&M&C	Pro Taper	X4	AH Plus^c^
Bolles et al. [10]	Mandibular premolars	Nail Polish	6.0% NaOCl & 17% EDTA	× 5	SI(15), CNI(15)	A&C	Flexo File	40/.06	Simpli Sealer^d^
Ch et al. [26]	Mandibular premolars	Glue	5.25% NaOCl & 17% EDTA	× 10	SI(19), CNI(19)	A&M&C	Pro Taper	F4	AH Plus^a^
Generali et al. [27]	Mandibular premolars	Cyanoacrylate	5.25% NaOCl &17% EDTA	× 5 & × 10	SI(10), CNI(10)	A&M&C	Pro Taper	F4	AH Plus^a^
Machado et al. [28]	Maxillary molar	Unknown	5.0% NaOCl & 17% EDTA	Unknown	SI(13), CNI(13)	A&C	Pro Taper	F4	AH Plus^a^
Uğur Aydin et al. [17]	Maxillary incisors	Unknown	2.5% NaOCl & 17% EDTA	× 10	SI(15), CNI(15)	A&M&C	Pro Taper	F4	AH Plus^a^
Yilmaz et al. [29]	Maxillary molar	Unknown	5.25% NaOCl & 17% EDTA	× 10	SI(15), CNI(15)	A&C	Pro Taper	X3	AH Plus^a^

A&M&C respectively represents apical, middle, coronal third of root canal; Superscript a&b&c&d respectively represents different brands (DentsplyDeTrey, Konstanz, Germany), (Brasseler USA, Savannah, GA), (Dentsply, Ballaigues, Switzerland), (Discus Dental, Culver City, CA)

### Data synthesis

A narrative synthesis was performed for all included studies screened by inclusion criteria, while a meta-analysis was confined to results that were quantitatively presented in the form of means and standard deviations, or in the form of enabling manual calculation (i.e., frequency tables) through Excel 2010 (Microsoft Corporation, Washington, USA). For the study in which the data in the outcomes are presented as the median, minimum and maximum values and the first and third quartiles, the method of [21] was used to convert those data from the reported summary data into the mean or standard deviation for analysis. If all the above methods failed to obtain raw data, then raw data were requested from principal authors by sending an e-mail. ImageJ 1.38e software (Wayne Rasband, National Institutes of Health, USA) was used to obtain raw data that were presented in the form of graphs and not provided by the author of the included studies. For the three different regions of the root canal (we defined the different sections (apical, middle, coronal) based on the description of the included study itself), a comprehensive meta-analysis including all studies was performed for SI with respect to CNI. Since all the measurement indices included in the studies such as the mean and standard deviation of percentage (%) and maximum depth (µm) of sealer penetration, have the same measurement units, the weighted mean difference (WMD) was used to compare these variables. Outcomes are shown in forest plots where the edges and middle of the rhombus represent the 95% confidence interval (95% CI) and the WMD point estimate, respectively. The 95% CI and point estimate for each study are presented as a horizontal line and a central symbol, respectively. Chi-squared analyses and I^2^ scores were displayed to analyse homogeneity. Random-effects models were used for the meta-analysis. All calculations were carried out using Review Manager 5.4.

### Risk of bias assessment

The critical estimative checklist of randomized controlled trials of the standardized Joanna Briggs Institute (JBI) was adopted to independently score the quality of each study. As modified by Felipe et al. [22], this key appraisal tool is suitable for evaluating CLSM experimental studies. This tool has a total of 13 questions, each of which can be answered yes, no, or unclear. The following issues were used in the evaluation: (Q.1) Was the teeth capacity calculated? (Q.2) Was randomization designed when assigning teeth to SI and CNI groups? (Q.3) Was the investigator blinded to the allocation of the SI and CNI groups? (Q.4) Were the characteristics of the SI and CNI groups similar at baseline? (Q.5) Were the experimental operations of SI and CNI groups completed by the same person? (Q.6) Were those performing CNI and SI on tooth samples blinded to the groups design? (Q.7) Were SI and CNI treated equally except the intervened variables? (Q.8) Were the data items of the studies screened by the designated reviewer? (Q.9) Was the outcome evaluator blinded to the group design? (Q.10)Were the outcomes of the studies assessed by the same method for SI and CNI? (Q.11) Was the statistical analysis method used in the studies reasonable? (Q.12) Were study results reporting all tooth samples? If not, were the reasons for not reporting explained? and (Q.13) Was the design of the experiment reasonable without other biases? If the details of the report are insufficient to properly answer the questions, the judgement is considered ‘unclear’. The bias risk of the study was classified as ‘high’, ‘moderate’ and ‘low’ when the number of yes answers was less than or equal to 5, between 6 and 8, and greater than or equal to 8, respectively. All data are summarized in Table 7.

### Publication bias

Since only 8 articles were selected to conduct the meta-analysis, it is not justifiable to use funnel plots and related statistical tests for analysis, as tests for publication bias only have sufficient power when there are at least 10 studies (Higgins JPT, Green S, editors (2009) Cochrane Handbook for Systematic Reviews of Interventions Version 5.0.2. The Cochrane Collaboration. Available at www.cochrane-handbook.org. Accessed May 10, 2010).

### Sensitivity analysis

Sensitivity analysis was performed by omitting each study from the meta-analysis until heterogeneity decreased significantly. If there was no difference in the meta-analysis synthesis results before and after excluding the relevant literature, then it proves that the original synthesis results were relatively stable.

### Certainty of evidence

The GRADEprofiler software (Version 3.6, GRADE Working Group) was used to assess the certainty of the evidence. The quality of the evidence can be downgraded by five domains (inconsistency, risk of bias, imprecision, indirectness, other considerations) [23]. All of outcomes are shown in Table 8.

## Results

### Selected studies

**Fig. 1:**
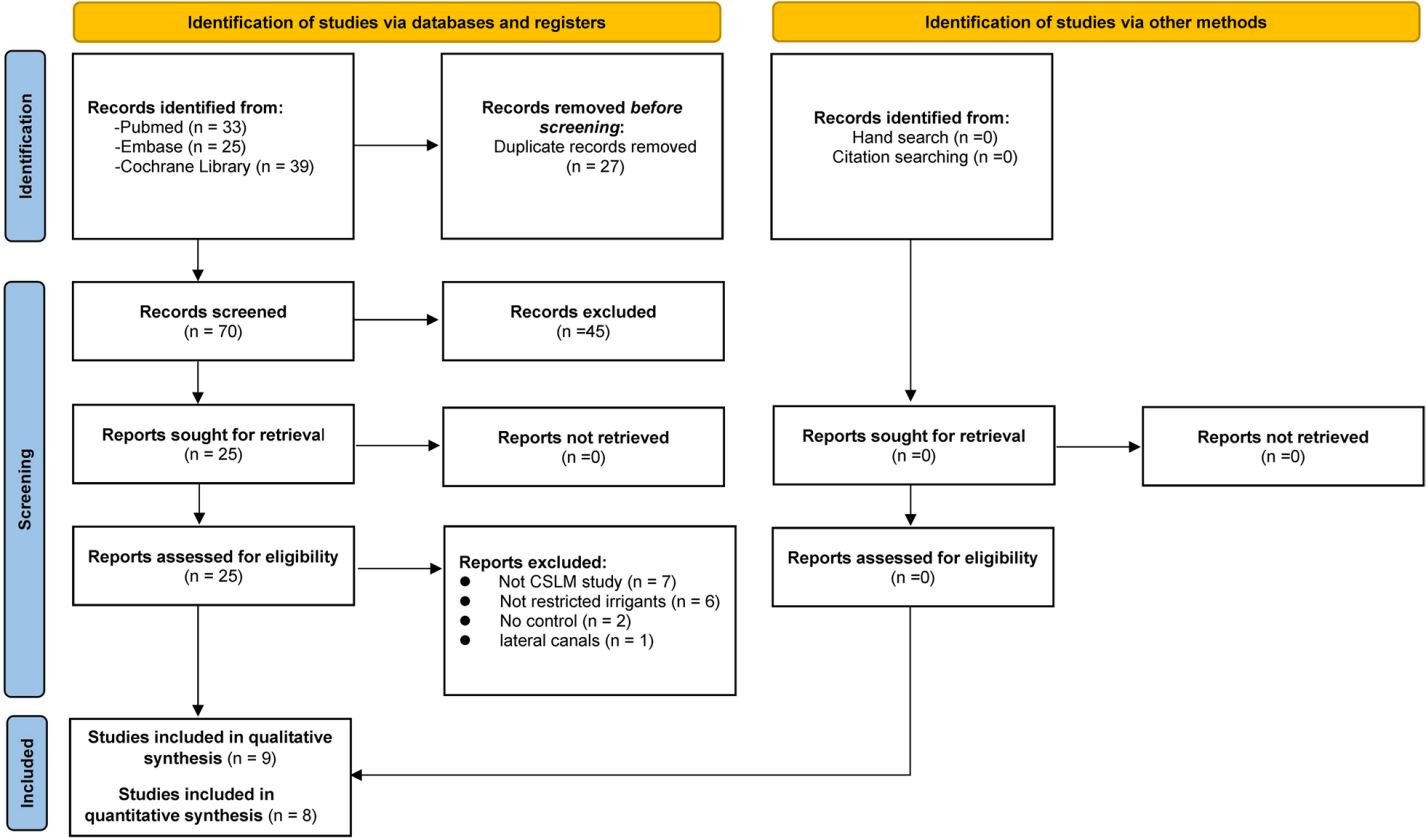
Flow diagram based on PRISMA 2020 guidelines

From the initial search, 97 studies were selected in total. Of those 27 were eliminated because of duplications and 70 of the remaining studies were judged according to the inclusion criteria (Fig. 1). Twenty-five studies met the conditions for full-text review after being subjected to title and abstract screening, of which 9 directly made comparisons between CNI and SI under the previously mentioned criteria, making them eligible for inclusion (Table 2). Only 8 of those studies provided a meta-analysis with enough quantitative data [10, 17, 24–29]. Table 3 provides a detailed explanation of why the 16 publications were rejected and excluded from the full-text review.

**Table 3:** Disqualification reasons of articles

Article	Disqualification reasons	
1	Khaleel et al. [47]	Not an in vitro CLSM study
2	Iandolo et al. [48]	Not an in vitro CLSM study
3	Tungsawat et al. [49]	Not an in vitro CLSM study
4	Koruk et al. [50]	Irrigants is not NaOCl & EDTA
5	Keskin et al. [51]	Irrigants is not NaOCl & EDTA
6	Salas et al. [52]	Irrigants is not NaOCl & EDTA
7	Matos et al. [53]	Irrigants is not NaOCl & EDTA
8	Bernabé et al. [44]	Not an in vitro CLSM study
9	Arslan et al. [54]	Lateral canals
10	Nikhil et al. [55]	No CNI control group
11	Chaudhry et al. [56]	No CNI control group
12	Oliveira et al. [18]	Not an in vitro CLSM study
13	Klyn et al. [57]	Not an in vitro CLSM study
14	Virdee et al. [58]	Not an in vitro CLSM study
15	Özlek, et al. [59]	Irrigants is not NaOCl & EDTA
16	Küçük et al. [60]	Irrigants is not NaOCl & EDTA

### Study characteristics

#### Teeth sample

Nine articles adopted the CLSM method for research, and all of them were published between 2010 and 2022. The most frequently studied teeth were mandibular premolars (n = 5), followed by the maxillary molars (n = 2) and then the maxillary and mandibular incisors (n = 2) (Table 2). The root canal system was closed at the apical position using cyanoacrylate (n = 3), wax (n = 2) or nail polish (n = 1), and three articles did not reveal details about whether the system was closed. Teeth were irrigated with NaOCl concentrations ranging between 2.50–5.25% and 17% EDTA (Table 2).

#### Details of using CNI

Conventional needle irrigation (n = 9): The classification of needles applied included side vented (n = 4) and open ended (n = 5) with sizes of 30 G (n = 5), 31 G (n = 1), 29 G (n = 1), 28 G (n = 1) and 27 G (n = 1). The depth of needle insertion from the working length was determined to be 1 mm (n = 4) or 2 mm (n = 4). The irrigation time was 1 to 2 min. The insertion depth of one study was unclear because no related details could be found (Table 4).

**Table 4:** Details of using conventional needle irrigation

Study		Details of using conventional needle irrigation				
		AT (s)	Manufacturer	End Type	Gauge	Depth from WL
Akcay et al. [30]		60	NaviTip	Open-Ended	27	1 mm
Ateş et al. [24]		120	NaviTip	Side-Vented	30	1 mm
Bharti et al. [25]		60	NaviTip	Open-Ended	30	Unknown
Bolles et al. [10]		60	Dentsply	Side-Vented	30	1 mm
Ch et al. [26]		120	Max-i-Probe	Side-Vented	28	2 mm
Generali et al. [27]		90	Dentsply	Side-Vented	30	2 mm
Machado et al. [28]		120	NaviTip	Open-Ended	29	2 mm
Uğur Aydin et al. [17]		90	NaviTip	Open-Ended	31	1 mm
Yilmaz et al. [29]		120	NaviTip	Open-Ended	30	2 mm

*AT* represents Agitation time, *WL* represents working length

#### Details of using SI

Sonic irrigation (n = 9): The set value of power for SI was at 10 000 cycles per minute (n = 5). The most common taper size was 25.04 (n = 7) followed by size 35.04 (n = 1) and 15.02 (n = 1) taper tips. The insertion depth of the needle from the working length was determined to be 2 mm (n = 7) or 1 mm (n = 2) with irrigation time fluctuating from 1 to 2 min. Four studies did not mention information about the set value of power (Table 5).

**Table 5:** Details of using sonic irrigation techniques

Study	Details of using Sonic irrigation techniques				
	AT (s)	System	Power setting (cycles/min)	Tips	Depth from WL (mm)
Akcay et al. [30]	60	EndoActivator	10,000	25/.04	2
Ateş et al. [24]	120	EndoActivator	10,000	25/.04	2
Bharti et al. [25]	60	EndoActivator	10,000	15/.02	2
Bolles et al. [10]	60	EndoActivator	Unknown	25/.04	2
Ch et al. [26]	120	EndoActivator	Unknown	25/.04	2
Generali et al. [27]	90	EndoActivator	Unknown	25/.04	2
Machado et al. [28]	120	EndoActivator	10,000	35/.04	1
Uğur Aydin et al. [17]	90	EDDY	Unknown	25/.04	1
Yilmaz et al. [29]	120	EndoActivator	10,000	25/.04	2

*AT* represents Agitation time, *WL* represents working length

#### CLSM evaluation

Eight studies investigated both the maximum depth and percentage of sealer penetration. In addition, one exclusively investigated the percentage of sealer penetration. All 9 studies assessed the apical and coronal segments of canals, while fewer studies evaluated the middle third (n = 5) (Table 2). The CLSM magnification was used × 5 (n = 2), × 10 (n = 4) and × 5 & × 10 (n = 1) for tubular dentin sealer penetration (Table 2). Two studies did not disclose details on CLSM magnification.

#### Statistical methods

The statistical methods adopted to evaluate the significance of the maximum depth and percentage of sealer penetration for SI and CNI included the Kruskal–Wallis test with post hoc analysis [10, 24, 25, 27, 29], and ANOVA test followed by post hoc analysis [26, 28, 30].

### Meta-analysis

#### SI versus CNI in the apical region

**Fig. 2:**
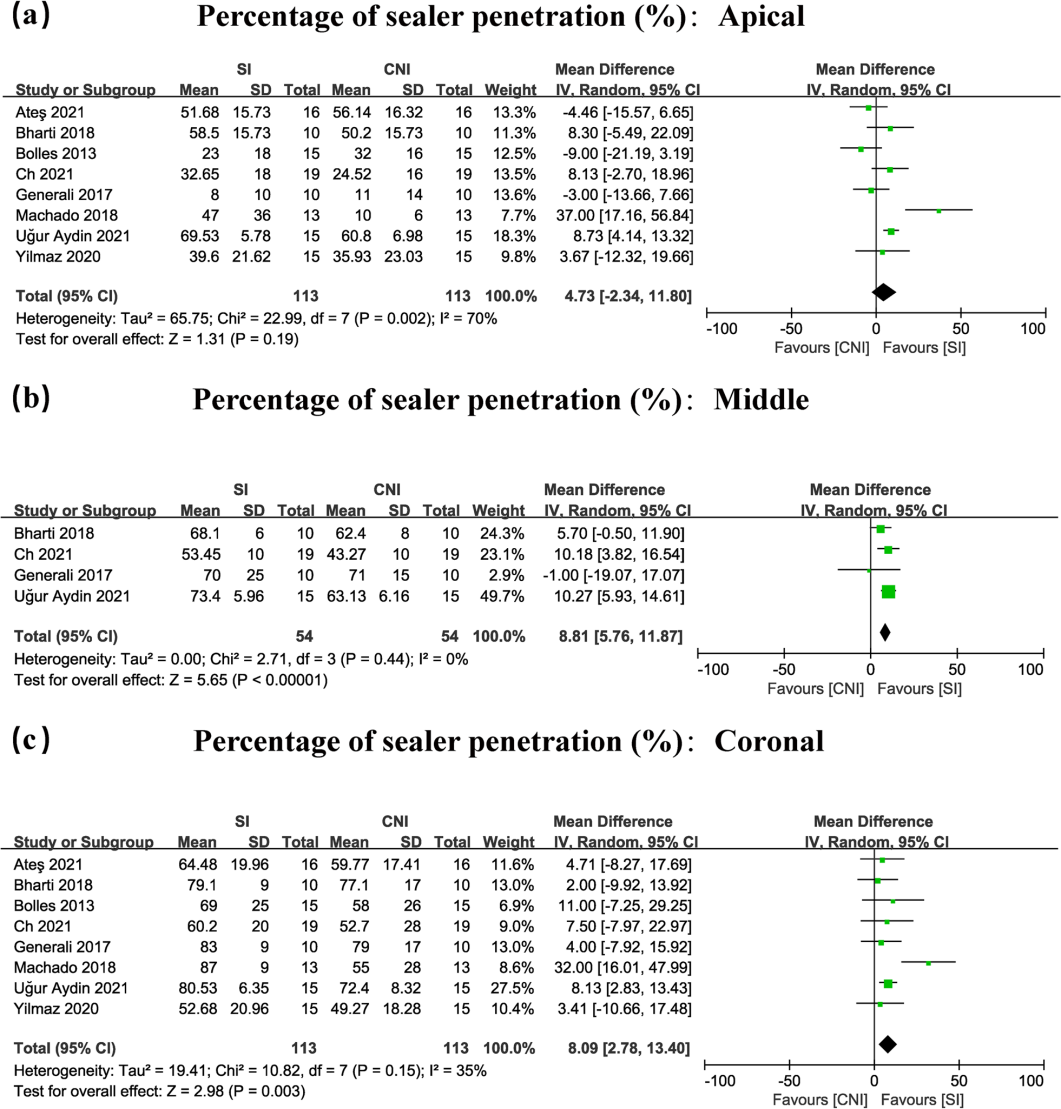
Forest plots for percentage of sealer penetration in the apical (**a**), middle (**b**), coronal (**c**) of root canals comparing the use of SI with CNI

**Fig. 3:**
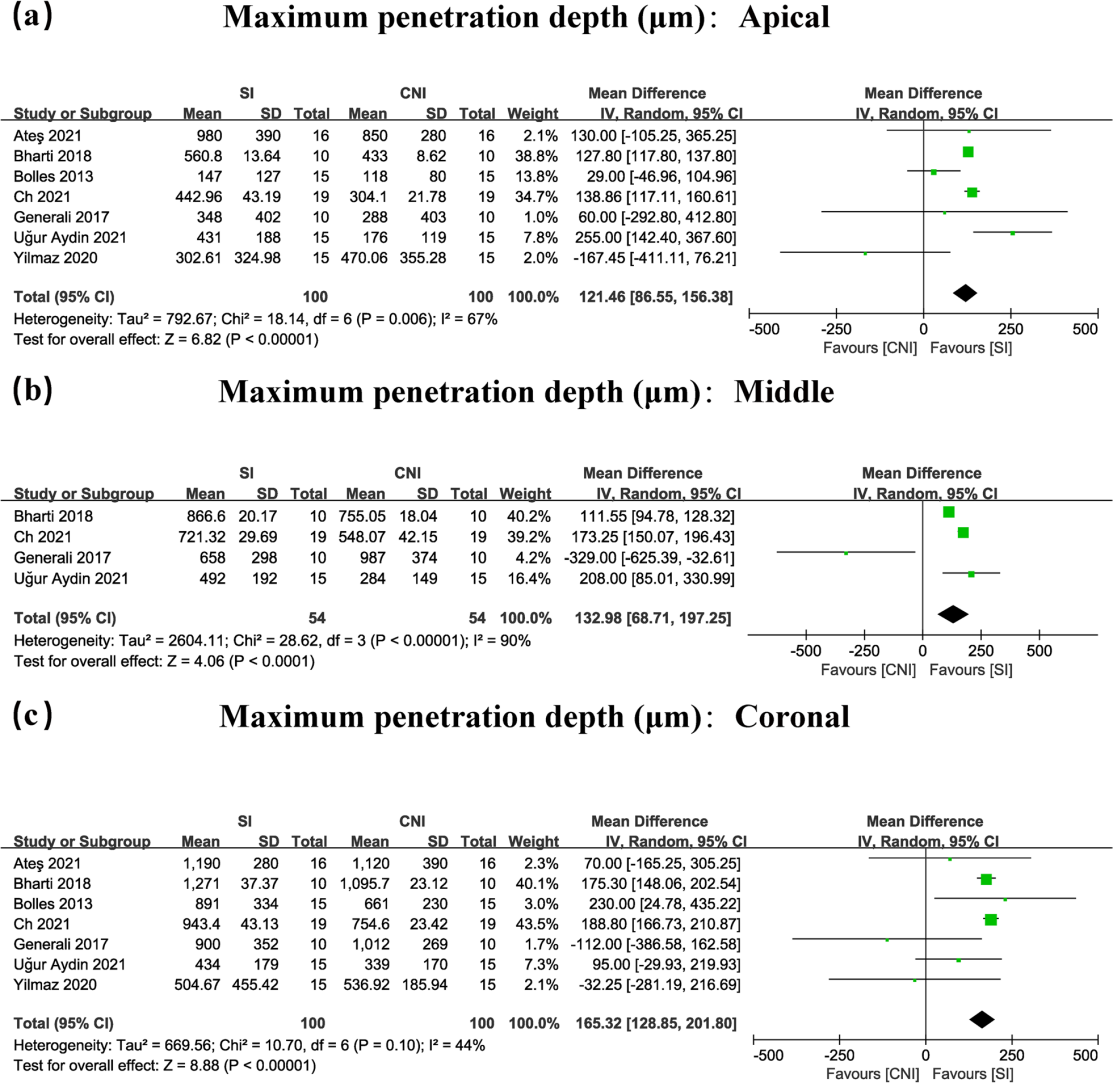
Forest plots for maximum depth of sealer penetration in the apical (**a**), middle (**b**), coronal (**c**) of root canals comparing the use of SI with CNI

The statistical data showed that the percentage of sealer penetration in the apical thirds region was nonsignificant (WMD: 4.73, 95% CI − 2.34–11.80) (Figs. 2a, 3a). However, the maximum depth of sealer penetration in the apical thirds region was important (WMD: 121.46, 95% CI 86.55–156.38) (Figs. 2a, 3a).

#### SI versus CNI in the middle region

The meta-analysis demonstrated significant improvements in the middle region (WMD: 8.81, 95% CI 5.76–11.87/WMD: 132.98, 95% CI 68.71–197.25) (Figs. 2b, 3b), for percentage and maximum depth of sealer penetration, respectively.

#### SI versus CNI in the coronal region

The results showed substantial improvements in the coronal region (WMD: 8.09, 95% CI 2.78–13.40/WMD: 165.32, 95% CI 128.85–201.80) (Figs. 2c, 3c), for the percentage and maximum depth of sealer penetration, respectively.

The outcomes from all studies limited to quantitative synthesis (meta-analysis) are given in Table 6.

**Table 6:** Summary of the parameters included in the meta-analysis

Authors	GA	Coronal (M ± SD)		Middle (M ± SD)		Apical (M ± SD)	
		Percentage (%)	Maximum (μm)	Percentage (%)	Maximum (μm)	Percentage (%)	Maximum (μm)
Ateş et al. [24]	SI	64.48 ± 19.96	1190 ± 280			51.68 ± 15.73	980 ± 390
	CNI	59.77 ± 17.41	1120 ± 390			56.14 ± 16.32	850 ± 280
Bharti et al. [25]	SI	79.1 ± 9.0	1271 ± 37.37	68.1 ± 6	866.6 ± 20.17	58.5 ± 15.73	560.8 ± 13.64
	CNI	77.1 ± 17	1095.7 ± 32.12	62.4 ± 8	755.05 ± 18.04	50.2 ± 15.73	433 ± 8.62
Bolles et al. [10]	SI	69 ± 25	891 ± 334			23 ± 18	147 ± 127
	CNI	58 ± 26	661 ± 230			32 ± 16	118 ± 80
Ch et al. [26]	SI	60.2 ± 20	943.4 ± 43.13	53.45 ± 10	721.32 ± 29.69	32.65 ± 18	442.96 ± 43.19
	CNI	52.7 ± 28	754.6 ± 23.42	43.27 ± 10	548.07 ± 42.15	24.52 ± 16	304.1 ± 21.78
Generali et al. [27]	SI	83 ± 9	900 ± 352	70 ± 25	658 ± 298	8 ± 10	348 ± 402
	CNI	79 ± 17	1012 ± 269	71 ± 15	987 ± 374	10 ± 11	288 ± 403
Machado et al. [28]	SI	87 ± 9				47 ± 36	
	CNI	55 ± 28				10 ± 6	
Uğur Aydin et al. [17]	SI	80.53 ± 6.35	434 ± 179	73.4 ± 5.96	492 ± 192	69.53 ± 5.78	431 ± 188
	CNI	72.4 ± 8.32	339 ± 170	63.13 ± 6.16	284 ± 149	60.8 ± 6.98	176 ± 119
Yilmaz et al. [29]	SI	52.68 ± 20.96	504.67 ± 455.42			39.6 ± 21.62	302.61 ± 324.98
	CNI	49.27 ± 18.28	536.92 ± 185.94			35.93 ± 23.03	470.06 ± 355.28

GA represents group allocation; Empty parts indicate a lack of information for the corresponding researchs

### Heterogeneity tests

When analysing the meta-analysis of the percentage of sealer penetration with 8 articles included, the chi-squared tests showed that there was notable heterogeneity in the apical region (I^2^ = 70%, *P* = 0.002). However, sufficient homogeneity was determined in the middle (I^2^ = 0%, *P* = 0.44), and coronal (I^2^ = 35%, *P* = 0.15) portions of the canal (Fig. 2a, b, c).

When analysing the meta-analysis in maximum depth of sealer penetration with 7 articles included, the chi-squared tests showed that there was significant heterogeneity in the apical (I^2^ = 67%, *P* = 0.006) and middle (I^2^ = 90%, *P* < 0.00001). However, sufficient homogeneity was found in the coronal (I^2^ = 44%, *P* = 0.10) portions of the canal (Fig. 3a, b, c).

### Bias assessment

**Fig. 4:**
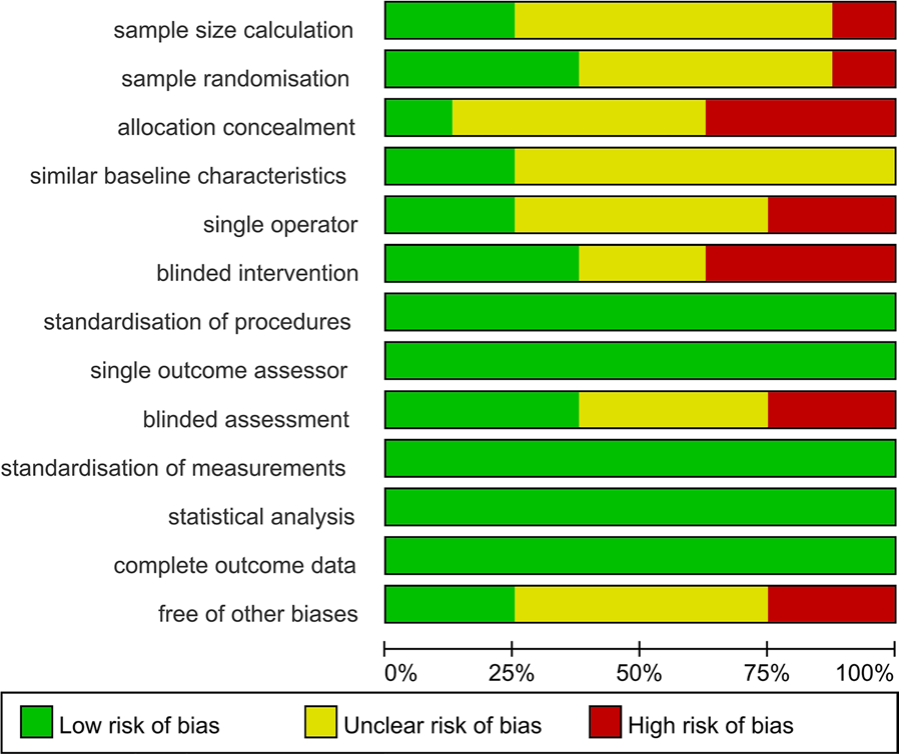
A summary of each risk of bias item as percentages across all included studies

Table 7 provides conclusions after a review of the individual risk of bias items and methodological quality of the each article that met the inclusion criteria of the meta-analysis. Four studies [10, 17, 27, 30] presented a low risk of bias, and four studies presented a moderate risk of bias [24, 25, 28, 29]. Only one study presented a high risk of bias [26]. All of the items of risk of bias are summarized in Fig. 4 in the form of percentages across these studies.

**Table 7:** Risk of bias and individual quality of the studies

Authors	Q.1	Q.2	Q.3	Q.4	Q.5	Q.6	Q.7	Q.8	Q.9	Q.10	Q.11	Q.12	Q.13	‘Yes’(n)	Risk of bias
Akcay et al. [30]	U	Y	U	Y	U	Y	Y	Y	N	Y	Y	Y	Y	9	Low
Ateş et al. [24]	U	U	U	U	U	N	Y	Y	Y	Y	Y	Y	U	6	Moderate
Bharti et al. [25]	N	Y	U	U	N	Y	Y	Y	N	Y	Y	Y	U	7	Moderate
Bolles et al. [10]	Y	U	U	Y	Y	N	Y	Y	Y	Y	Y	Y	Y	10	Low
Ch et al. [26]	U	N	N	U	U	U	Y	Y	N	Y	Y	Y	U	5	High
Generali et al. [27]	U	Y	U	Y	U	Y	Y	Y	U	Y	Y	Y	U	8	Low
Machado et al. [28]	U	U	Y	U	U	N	Y	Y	Y	Y	Y	Y	N	7	Moderate
Uğur Aydin et al. [17]	Y	U	N	U	Y	U	Y	Y	U	Y	Y	Y	Y	8	Low
Yilmaz et al. [29]	U	Y	N	U	N	Y	Y	Y	U	Y	Y	Y	N	7	Moderate

Questions 1 to 13 are described in detail in ‘Risk of bias assessment’ of the method section. Yes/No/Unclear respectively represented by “Y”, “N”, “U”. The bias risk of the study was respectively classifified as ‘high’, ‘moderate’ and ‘low’, when the number of yes is less than or equal to 5, between 6 and 8, and greater than or equal to 8

### Sensitivity analysis

The conclusions of the sensitivity analysis are shown in Additional file 1: Figure S1 and Additional file 2: Figure S2.

The sensitivity analysis was performed by omitting each study from the meta-analysis until sufficient homogeneity was achieved (I^2^ = 0%, *P* ranged from 0.42 to 0.95). After excluding the relevant studies, the meta-analysis demonstrated significant improvements in the coronal (WMD: 6.50, 95% CI 2.67–10.34/WMD: 181.65, 95% CI 164.68–198.45), middle (WMD: 8.81, 95% CI 5.76–11.87/WMD: 174.44, 95% CI 151.66–197.22), and apical regions (WMD: 8.31, 95% CI 4.40–12.23/WMD: 218.56, 95% CI 120.96–316.16) for percentage and maximum depth of sealer penetration, respectively. All of the results in the original synthesis were relatively stable except for the maximum depth of sealer penetration in the apical third region when compared with the outcomes of the meta-analysis after excluding the relevant studies.

### Certainty of the evidence

The judgements of the GRADE assessment are shown in Table 8.

**Table 8:** Certainty of the evidence from included studies based on GRADEprofiler

Comparison	Number of studies	Study design	Risk of bias	Inconsistency	Indirectness	Imprecision	Other considerations	Certainty
Certainty assessment								
P&A	Eight	RCT	Serious^a^	Serious^b^	No	No	No	LOW
P&M	Four	RCT	Serious^a^	No	No	No	No	MODERATE
P&C	Eight	RCT	Serious^a^	No	No	No	No	MODERATE
MD&A	Seven	RCT	Serious^a^	Serious^b^	No	No	No	LOW
MD&M	Four	RCT	Serious^a^	Serious^b^	No	No	No	LOW
MD&C	Seven	RCT	Serious^a^	No	No	No	No	MODERATE

P&A, P&M, and P&C represent percentage of sealer penetration in the apical, middle, and coronal third respectively

MD&A, MD&M and MD&C represent maximum depth of sealer penetration in the apical, middle, and coronal third respectively

^a^All studies included had a bias accroding to Table 7

^b^High heterogeneity (I^2^ ˃ 50%) has been shown accroding to (Fig. 2a–c) & (Fig. 3a–c)

The certainty of the evidence was low in the studies comparing SI versus CNI in the apical region for both percentage and maximum depth of sealer penetration; The certainty of the evidence was moderate in the studies comparing SI versus CNI in the middle region for percentage of sealer penetration and was low in the studies comparing SI versus CNI in the middle region for maximum depth of sealer penetration; The certainty of the evidence was moderate in the studies comparing SI versus CNI in the coronal region for both percentage and maximum depth of sealer penetration.

## Discussion

In general, the results show that compared with CNI, SI greatly increased tubular dentin sealer penetration across a large region of the root canal. Sufficient homogeneity was determined in the coronal third region in both the percentage and maximum depth of sealer penetration (I^2^ = 35%, *P* = 0.15/I^2^ = 44%, *P* = 0.10) (Figs. 2c, 3c). We can confirm that SI produces better tubular dentin sealer penetration in the coronal region of the root canal than CNI. However, very large heterogeneity was found in the apical third of the canal in both the percentage and maximum depth of sealer penetration (I^2^ = 70%, *P* = 0.002/I^2^ = 67%, *P* = 0.006). The significant heterogeneity of the study of the apical third region suggests that we need more standardized data on this region to conduct a more accurate analysis in the future. After reviewing the excluded studies in the sensitivity test, we found that the reason for high heterogeneity may be the lack of standardization in methodology in the whole process of root canal filling. For example, most studies included in this article were carried out on straight root canals of the teeth, but the other study, which was excluded in the sensitivity test of the apical third region (Additional file 2: Figure S2a) used teeth with curved root canals [29]. Before and after excluding this study, the percentage of sealer penetration in the apical third region changed from nonsignificant to significant (WMD: 4.73, 95% CI − 2.34–11.80/WMD: 8.31, 95% CI 4.40–12.23) (Figs. 2a, Additional file 2: S2a). The main possible reason for the unstable outcome in this region was that curved root canals would make irrigation solution difficult to transport to the apical region of the root canal even using the SI technique [31]. Therefore, based on the sensitivity test results of the apical third region (Figs. 2a & Additional file 2: S2a), we believe that curved root canals may be one of the obstacles to achieving high apical sealer penetration in clinical practise. Moreover, there are other reasons that may have a very large effect on the penetration of sealers, including the diameter of the root canal, the type of irrigants, the concentration and volume of the irrigants, obturation techniques, and the different filling materials [13, 32–34]. These factors may also become one of the possible reasons why some studies have reported that SI produces no obvious contrast in tubular dentin sealer penetration compared with CNI in the apical third of the root canal [10, 24, 27, 29]. Thus, in the future, it is necessary to standardize root canal irrigation procedures and provide more accurate results in this area. In addition, the apical region has fewer dentinal tubules with smaller diameters, more sclerotic dentin, and more difficult access for sealing the dentinal tubules [35, 36], which may become the other important explanation why in some studies, there is no sealer penetration difference found in the apical region between SI and CNI. This also implies that the sealer penetration efficiency of SI in the apical region needs to be improved in the future according to the anatomical structure of the root canal in this area.

To identify possible confounding factors in our study, we further found that, compared with other studies using epoxy resin-based sealers (AH Plus, Simpli Sealer), the included study [24] used bioceramic-based sealers (BC sealer). This is a parameter that may affect sealer penetration due to its physicochemical character. However, because some studies note that the flow rates of BC sealer and AH Plus are similar (*p* > 0.05), and that the flow rate has a very large impact on sealer penetration, the similarity of character between the two sealers makes them have similar sealer penetration [37, 38]. Therefore, we believe that the inclusion of this study [24] is reasonable and will not be a possible confounding factor in our study. Moreover, other studies that investigated the penetration of bioceramic sealers and used AH Plus sealer as a control group found that there was no significant difference in sealer penetration between the two types of sealers [39, 40], which also supported our inclusion of the study involving BC sealer. The use of the different systems of sonic activation (EndoActivator, EDDY) in our study may become the other possible confounding factor. However no study has directly compared the efficiency of the two sonic activation techniques in the sealer penetration. Some studies have shown that EndoActivator and EDDY perform equally efficiently in debris and smear layer removal [41, 42]. Therefore, we believe this parameter may not be a possible confounding factor in our study because the residual smear layer can greatly affect the sealer penetration by adhereing to the surface of dentin tubules and preventing sealers from entering the dentin tubules [9]. The incomplete information of the included studies may also become a confounding factor (the insertion depth of one study was unclear, four studies did not mention information about the set value of power, and two studies did not disclose details on CLSM magnification). Therefore, the heterogeneity of the apical region may be partially caused by this confounding factor, and in the future, more relevant and detailed research is needed to reduce heterogeneity and accurately reveal the sealer penetration efficiency of SI in the apical region.

On the other hand, even if there is heterogeneity of the apical third region, which leads to a lack of a precise conclusion in this region in meta-analysis, the results of narrative synthesis really showed that, compared with CNI, SI has greater sealer penetration in the coronal and middle third regions of the root canal, as all studies included in the meta-analysis concluded significant improvements in the percentage and maximum depth of sealer penetration (Fig. 2b, c and Fig. 3b, c) following irrigant agitation. The SI is reported to have the following advantages: (1) This irrigation system has a stronger and unattenuated oscillation amplitude and frequency of tip than CNI which can accelerate the flow rate of irrigants during the irrigation and eliminate the vapor lock to improve sealer penetration [43]. (2) The tip of SI is made of highly flexible polyamide which is softer than the rigid metal tip of CNI and can largely avoid getting in touch with the canal walls when oscillating, which can maintain the original and unattenuated high amplitude of the tip in the process of use and lead to less production of the smear layer by not cutting the root canal dentin wall. Additionally, the less the smear layer that is produced, the better penetration the sealer will have [40, 41]. The above advantages of SI can also support the conclusion of this review which verified that SI greatly strengthens tubular dentin sealer penetration across a large portion of the root canal, and because of that, this technique is suggested for root canal irrigation for its ability to better meet clinical demand which may lead to greater penetration and prevent reinfection [43, 44].

Finally, the outcomes of this systematic review are only founded on relevant research of CLSM, and different observation methods are eligible to study the sealer penetration, such as scanning electron microscopy (SEM), optical microscopy, and CLSM [45, 46]. Although CLSM has been proven to be the best method to estimate sealer penetration into dentinal tubules for the following reasons: (1) The CLSM has the highest detection accuracy and reduces the technical errors. (2) The CLSM does not require sample pretreatment. (3) CLSM can image optical sections without cleaning up the smear layer [24]. The lack of SEM and light microscope experiments may lead to insufficient data to support the conclusions of this study. Moreover, only two common indicators (the percentage and maximum depth of sealer penetration) chosen to study the sealing ability of sealers may overlook a part of existing studies and this current low level of evidence of included studies in evaluating the apical third region of tubular dentin sealer penetration suggests that further relevant research is needed in this area.

## Conclusion

This review verified that SI significantly improves tubular dentin sealer penetration across a large region of the canal. The data from the present study led to a rejection of the null hypothesis that there would be no differences in sealer penetration between the SI and CNI; thus, these findings imply that SI may lead to better filling efficiency and anti-reinfection effects than CNI during and after the root canal therapy. However, a large heterogeneity in the current data regarding the comparison of the sealing ability of SI versus CNI in the apical third region of the root canal was found, implying the necessity to standardize root canal irrigation procedures and obtain more accurate results in this area.

## Supplementary Information

**Additional file 1: Fig. S1 Forest plot of the sensitivity analysis for the percentage of sealer penetration.**

**Additional file 2: Fig. S2 Forest plot of the sensitivity analysis for the maximum depth of sealer penetration.**

**Additional file 3: Table.S1 PRISMA_2020_checklist.**

**Additional file 4: Table.S2 PRISMA_2020_abstract_checklist.**

**Additional file 5: Table.S3 Embase search strategy.**

**Additional file 6: Table.S4 Cochrane Library search strategy.**

## Data Availability

All data generated or analysed during this study are included in this published article.

## Abbreviations

SI: Sonic irrigation
CNI: Conventional needle irrigation
CLSM: Confocal laser scanning microscopy
WMD: Weighted mean difference
CI: Confidence intervals
GRADE: Grading of recommendations, assessment, development and evaluation
JBI: Joanna Briggs Institute
PRISMA: Preferred reporting items for systematic reviews and meta-analyses
SEM: Scanning electron microscope
AEJ: Australian Endodontic Journal
IEJ: International Endodontic Journal
JOE: Journal of Endodontics
