# Supplementary figures and images for: Comparison of sealer penetration of sonic activation versus conventional needle irrigation: a systematic review and meta-analysis of randomized controlled trials
Source: BMC Oral Health. 2022 Dec 3;22:566. doi: 10.1186/s12903-022-02608-1 (PMC9719620; doi:10.1186/s12903-022-02608-1)

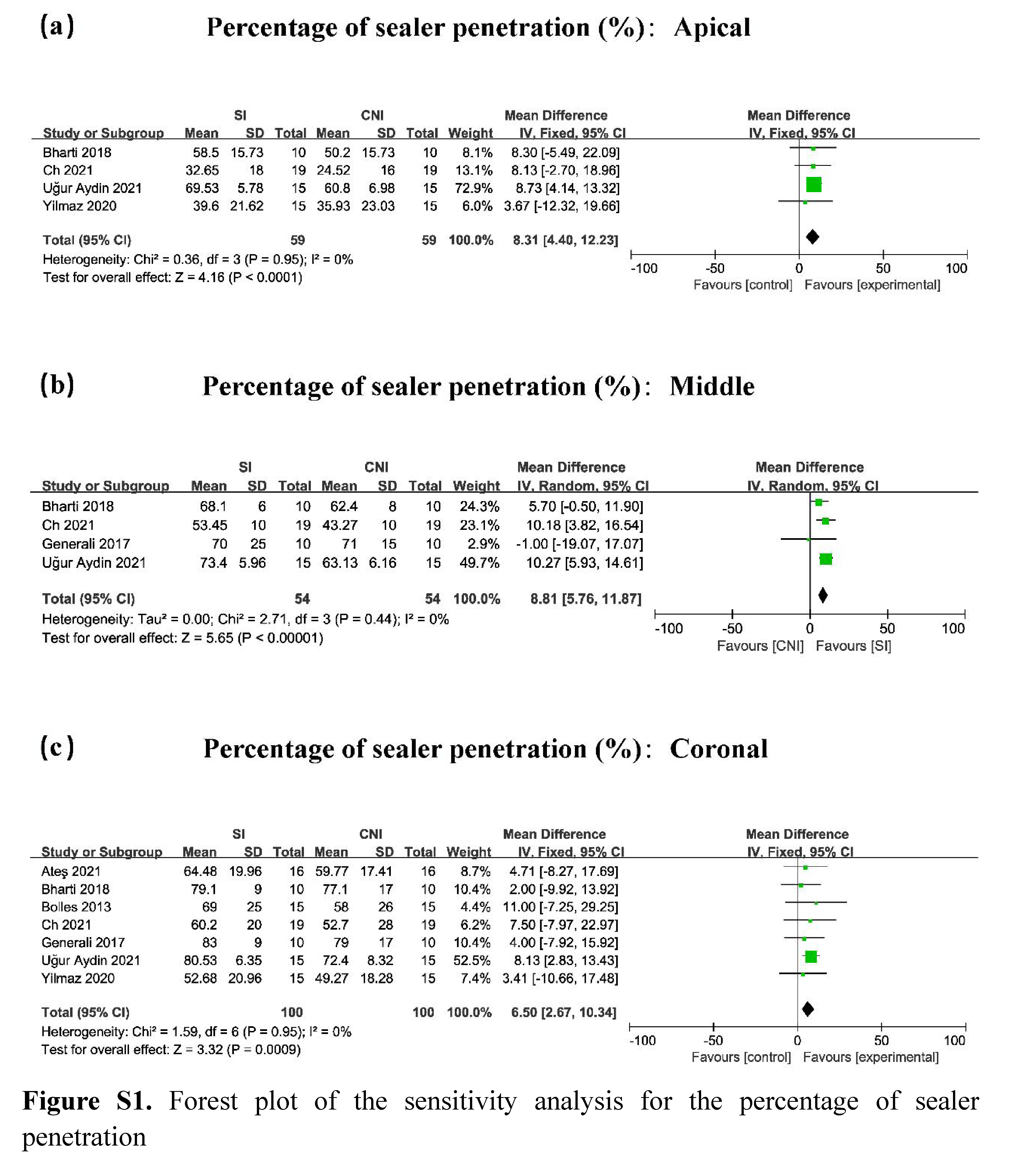

Supplement: Supplementary file 1 — Additional file 1: Fig. S1 Forest plot of the sensitivity analysis for the percentage of sealer penetration. [file 12903_2022_2608_MOESM1_ESM.tif]

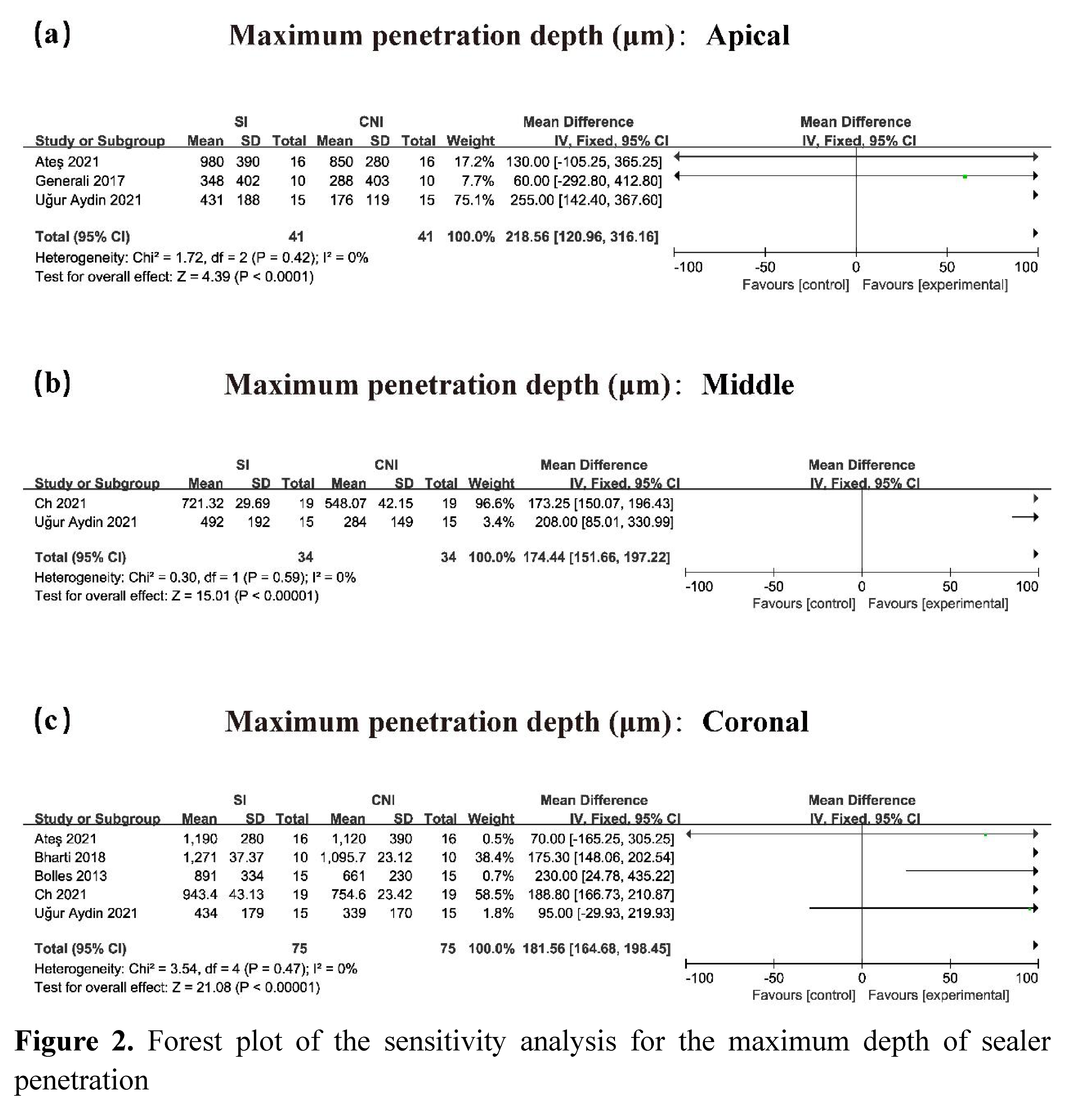

Supplement: Supplementary file 2 — Additional file 2: Fig. S2 Forest plot of the sensitivity analysis for the maximum depth of sealer penetration. [file 12903_2022_2608_MOESM2_ESM.tif]
