# Supplementary material for: Comparison of sealer penetration of sonic activation versus conventional needle irrigation: a systematic review and meta-analysis of randomized controlled trials
Source: BMC Oral Health. 2022 Dec 3;22:566. doi: 10.1186/s12903-022-02608-1 (PMC9719620; doi:10.1186/s12903-022-02608-1)
Supplement: Supplementary file 5 — Additional file 5: Table.S3 Embase search strategy. [file 12903_2022_2608_MOESM5_ESM.docx]

**Table S3** Embase search strategy

| Embase | | Search Strategy (October, 2022) | | | | | Items |
| --- | --- | --- | --- | --- | --- | --- | --- |
| #1 | 'dentinal tubules':ab,ti OR 'root canal':ab,ti OR 'root canals':ab,ti OR 'root dentine':ab,ti OR 'dentinal tubule':ab,ti OR premolars:ab,ti OR tubules:ab,ti OR dentition:ab,ti OR 'dentinal tubule'/exp OR 'dentition'/exp OR 'incisor'/exp OR 'premolar tooth'/exp OR 'canine tooth'/exp | | | | | |  |
|  |  |  |  |  |  |  | 293837 |
|  |  |  |  |  |  |  |  |
| #2 | 'sonic irrigation':ab,ti OR endoactivator:ab,ti OR sonication:ab,ti OR eddy:ab,ti OR 'sonic activation':ab,ti OR 'sonic'/exp | | | | | |  |
|  |  |  |  |  |  |  | 18681 |
|  |  |  |  |  |  |  |  |
| #3 | 'depth of penetration':ab,ti OR 'penetration depth':ab,ti OR 'sealer penetration':ab,ti OR sealing:ab,ti OR 'tubule penetration':ab,ti OR 'dentin permeability'/exp | | | | | |  |
|  |  |  |  |  |  |  | 21616 |
|  |  |  |  |  |  |  |  |
| #4 |  |  |  |  |  |  |  |
|  | #1 AND #2 AND #3 | | | | | | 25 |
|  |  |  |  |  |  |  |  |
