# Supplementary material for: Comparison of sealer penetration of sonic activation versus conventional needle irrigation: a systematic review and meta-analysis of randomized controlled trials
Source: BMC Oral Health. 2022 Dec 3;22:566. doi: 10.1186/s12903-022-02608-1 (PMC9719620; doi:10.1186/s12903-022-02608-1)
Supplement: Supplementary file 6 — Additional file 6: Table.S4 Cochrane Library search strategy. [file 12903_2022_2608_MOESM6_ESM.docx]

**Table S4** Cochrane Library search strategy

| Cochrane Library | | Search Strategy (October, 2022) | | | | | Items |
| --- | --- | --- | --- | --- | --- | --- | --- |
| #3: #1 or #2 | #1: (*sonic irrigation):ti,ab,kw or (endoactivator):ti,ab,kw or (sonication ):ti,ab,kw or (eddy):ti,ab,kw or (sonic activation):ti,ab,kw  #2: MeSH descriptor: [Sonication] explode all trees | | | | | |  |
|  |  |  |  |  |  |  | 976 |
|  |  |  |  |  |  |  |  |
| #6: #4 or #5 | #4: (depth of penetration):ti,ab,kw or (penetration depth):ti,ab,kw or (sealer penetration):ti,ab,kw or (sealing):ti,ab,kw or (tubule penetration):ti,ab,kw  #5: MeSH descriptor: [Dentin Permeability] explode all trees | | | | | |  |
|  |  |  |  |  |  |  | 8194 |
|  |  |  |  |  |  |  |  |
| #10: #7 or #8 or #9 | #7: (dentinal tubules):ti,ab,kw or (root canal):ti,ab,kw or (root canals):ti,ab,kw or (tubule):ti,ab,kw or (root dentine):ti,ab,kw  #8: (dentition):ti,ab,kw or (dentinal tubule):ti,ab,kw or (premolars):ti,ab,kw  #9:MeSH descriptor: [Dentition] explode all trees | | | | | |  |
|  |  |  |  |  |  |  | 14127 |
|  |  |  |  |  |  |  |  |
| #11 |  |  |  |  |  |  |  |
|  | #3 and #6 and #10 | | | | | | 39 |
|  |  |  |  |  |  |  |  |
